# Joint Effect of Genotypic and Phenotypic Features of Reproductive Factors on Endometrial Cancer Risk

**DOI:** 10.1038/srep15582

**Published:** 2015-10-26

**Authors:** Zhanwei Wang, Harvey Risch, Lingeng Lu, Melinda L. Irwin, Susan Mayne, Peter Schwartz, Thomas Rutherford, Immaculata De Vivo, Herbert Yu

**Affiliations:** 1 Cancer Epidemiology Program, University of Hawaii Cancer Center, Honolulu, HI; 2 Department of Chronic Disease Epidemiology, Yale School of Public Health, New Haven, CT, and Yale Cancer Center; 3 Department of Obstetrics and Gynecology, Yale School of Medicine, New Haven, CT, and Yale Cancer Center; 4 Channing Division of Network Medicine, Department of Medicine, Brigham and Women’s Hospital and Harvard Medical School, Boston, MA 02115, USA; 5 Department of Epidemiology, Program in Genetic Epidemiology and Statistical Genetics, Harvard School of Public Health, Boston, MA 02115, USA

## Abstract

Prolonged estrogen exposure is believed to be the major cause of endometrial cancer. As possible markers of estrogen exposure, various menstrual and reproductive features, e.g., ages at menarche and menopause, are found to be associated with endometrial cancer risk. In order to assess their combined effects on endometrial cancer, we created the total number of menstrual cycles (TNMC) that a woman experienced during her life or up to the time of study and two genetic risk scores, GRS1 for age at menarche and GRS2 for age at menopause. Comparing 482 endometrial cancer patients with 571 population controls, we found TNMC was associated with endometrial cancer risk and that the association remained statistically significant after adjustment for obesity and other potential confounders. Risk increased by about 2.5% for every additional 10 menstrual-cycles. The study also showed that high GRS1 was associated with increased risk. This relationship, however, was attenuated after adjustment for obesity. Our study further indicated women with high TNMC and GRS1 had twice the risk of endometrial cancer compared to those low in both indices. Our results provided additional support to the involvement of estrogen exposure in endometrial cancer risk with regard to genetic background and lifestyle features.

Endometrial cancer is the most frequently diagnosed gynecological cancer in the developed countries, and the fourth-most common female malignancy after breast, lung and colorectal cancer^1^. The development of endometrial cancer has been linked to excessive exposure of the uterus to estrogens as well as to a relative hormone imbalance between estrogens and progesterone. Prolonged estrogen stimulation of endometrial cells can cause abnormal proliferation^2^, resulting in hyperplasia which can further progress to neoplastic transformation. Progesterone opposes the effect of estrogens by down-regulating the concentrations of estrogen receptors and promoting endometrial cell differentiation^3^,^4^.

Reproductive characteristics, such as parity and ages at menarche, menopause, and first live parturition, reflect, to a certain extent, exposure to endogenous estrogens and progesterone^5^. Epidemiologic studies have shown that early age at menarche, late age at menopause, and nulliparity are all associated with increased risk of endometrial cancer, whereas early age at first live birth and multiparity are related to reduced risk^6^,^7^,^8^,^9^,^10^,^11^,^12^. Collectively, some studies have used composite measures that incorporate several reproductive and menstrual features to investigate the inferred effects of estrogen exposure on health. For example, total number of menstrual cycles (TNMC) has been used to study associations of estrogen exposure with breast cancer^13^,^14^ and Alzheimer Disease^15^. No studies to-date have used TNMC to assess the relationship between estrogen exposure and endometrial cancer risk.

Common genetic variations have been suspected to be involved in endometrial cancer risk, but to date only one single nucleotide polymorphism (SNP), rs4430796 at 17q12 close to HNF1 homeoboxB (*HNFIB*), has been identified by genome-wide association study (GWAS)^16^,^17^. GWAS have also found a number of SNPs to be associated with ages at menarche^18^,^19^,^20^,^21^,^22^,^23^,^24^ and at natural menopause^23^,^24^,^25^,^26^. Genetic risk scoring (GRS) has been used to evaluate the effect of multiple SNPs on associated phenotypes. No study has examined reproductive GRS in relation to endometrial cancer risk. In the current study, we investigated independent and joint associations of a reproductive composite measure (TNMC) and GRS on endometrial cancer risk, with adjustment for other risk factors, such as obesity and sex hormone use.

## Results

### Characteristics of study subjects

Table 1 shows that the distributions of study variables between cases and controls. Compared to controls, cases completed fewer years of education (p = 10^−4.2^), were slightly younger (p = 0.053), more obese (p = 10^−26.8^), had a greater proportion whose BMI changed more than 35% (p = 10^−8.4^), were more likely to have had induced menopause (p = 10^−7.4^), were younger at menarche (p = 0.005), and were less likely to have used oral contraceptives (p = 0.010). Cases also experienced more menstrual cycles than controls, 422.9 versus 396.1 TNMC (p = 10^−4.3^), and had higher genetic risk scores for menarche, either non-weighted GRS1 (p = 0.021) or weighted GRS1 (p = 0.011). No difference was found in genetic risk score for menopause, either non-weighted GRS2 (p = 0.97) or weighted GRS2 (p = 0.92), nor was age at natural menopause significantly different between cases and controls (p = 0.68). In addition, cases and controls were not different in having a family history of cancer (p = 0.24) or use of estrogen (p = 0.074).

### Genetic risk scores for ages at menarche and natural menopause

From several GWAS reports, we identified for GRS calculations 26 menarche-associated and 22 menopause-associated SNPs that matched our selection criteria. Supplementary Table S1 lists the SNPs. Some of the reported SNPs did not exist in our genotyping data, and therefore proxy SNPs were used that had strong linkage disequilibrium with the reported SNPs. Using these SNPs, we calculated GRS1 for early menarche risk and GRS2 for late menopause risk. Correlations between GRS and ages at menarche and menopause are shown in Table 2. These scores had significant linear associations, (β = −0.27, p = 0.007) between GRS1 and age at menarche (i.e., higher score and younger age at menarche) and (β = 0.18, p = 0.009) between GRS2 and age at menopause (i.e., higher score and older age at menopause). These associations were similar when we used weighted GRS1 and GRS2 (Table 2).

### Associations of endometrial cancer with TNMC and GSRs

The associations of endometrial cancer risk with TNMC and GRS are shown in Table 3 for all study subjects. Higher TNMC (more menstrual cycles) or GRS1 (younger age at menarche) was associated with increased endometrial cancer risk. These associations were significant in a dose-response manner (continuous variables). Women with higher than median values of TNMC or GRS1 had 65% or 33% increased risk, respectively, in comparison to those with values lower than median (categorical variables). Controlling other confounders (age, race and education) and risk factors (BMI, OC and estrog en use) did not seem to affect associations with TNMC, but exogenous sex hormone use and BMI did affect the disease relationship with GRS1. GRS2 did not show any associations with endometrial cancer. To exclude the potential influence of induced menopause on our observed associations, we analyzed the data limited to women who had natural menopause (Table 4). The results showed little change for TNMC, though GRS1 was no longer significantly associated with disease risk. To control for the effect of obesity, we performed subgroup analysis on individuals stratified by BMI category (less than 25, between 25 and 30, and 30 or higher). Similar and significant associations were observed only in those who were overweight (see supplementary Tables S2, S3 and S4). These results did not change when weighted GRS1 was used in the analyses.

To assess whether TNMC and GRS1 have joint effects on endometrial cancer, we created a combined TNMC-GRS1 variable with four levels. Level one included those with low TNMC and low GRS1 (both less than their medians); level two was those with high GRS1 and low TNMC; level three with low GRS1 and high TNMC; and level four with high GRS1 and high TNMC (both higher than their medians). Table 5 shows the results of these analyses. A possible joint effect was indicated between the two factors although the interaction term in a logistic regression model was not statistically significant. Women with high numbers of total menstrual cycles plus high genetic risk scores for early-age menarche had 2-fold increased risk of endometrial cancer. This risk association was slightly attenuated after BMI or BMI change was adjusted in the analysis, but the relationship was still statistically significant. Using weighted GRS1 did not change the results appreciably.

## Discussion

Prolonged unopposed estrogen exposure is believed to be a key risk factor contributing to the development of endometrial cancer. Previous studies have investigated in endometrial cancer the roles of several major reproductive and menstrual features that are partially or potentially involved in estrogen exposure, such as age at menarche, age at first live parturition, parity number, age at last live parturition, and age at menopause. Individually, all of these factors have been found to be associated with risk of endometrial cancer^6^,^7^,^8^,^9^,^10^,^11^,^12^, but collectively these risk factors have not been studied together for their combined effect on risk of the disease. In this case-control study, we developed a composite measure that incorporated all of the key reproductive and menstrual features: total number of menstrual cycles experienced during life (or up to the time of the study). Our data showed that this new variable was significantly associated with endometrial cancer risk; higher numbers of menstrual cycles with higher risk of the disease. Quantitatively, each menstrual cycle adds 0.25% to higher endometrial cancer risk. Women with TNMC higher than the median number had 56% greater risk of endometrial cancer compared to women with TNMC less than the median. Although the association was slightly attenuated after adjusting for obesity, another substantial risk factor for endometrial cancer, the effect of TNMC remained statistically significant.

As a composite measure, the total number of menstrual cycles combines both reproductive and menstrual features. One of the most important hormonal changes during each menstrual cycle is the substantial rise and fall of estradiol levels^10^. Each menstrual cycle not only represents estrogenic stimulation of the uterus, but also reflects changes in balance between estrogens and progesterone. While early adolescent and late perimenopausal cycles tend to be anovulatory, earlier age at menarche and later age at menopause may still render a longer term of endometrial exposure to endogenous estrogens. Further, lesser parity (or nulliparity) increases the number of menstrual cycles, which also translates into more estrogenic stimulation. Thus, collectively the composite measure we developed, i.e., total number of menstrual cycles, suggests a relationship with cumulative estrogen exposure. Previous epidemiologic studies have shown that women with high numbers of menstrual cycles tend to have higher risk of breast cancer^13^,^14^. Our study shows that this positive association may also be true for endometrial cancer.

In addition to the use of a composite measure to assess the inferred phenotype of endogenous estrogen exposure, we also created two genetic measures, using genetic polymorphisms discovered by GWAS. Genetic risk scores were developed for their associations with endometrial cancer risk, one for age at menarche (GRS1) and one for age at menopause (GRS2). The method we used to construct genetic risk scores is well-established and widely employed by researchers in the genetic field^27^,^28^. One of the genetic risk scores, age at menarche (GRS1), was significantly associated with endometrial cancer risk, without adjustment. The association became insignificant after BMI and other factors were adjusted in the analysis, suggesting that such a genetic role in the disease risk may be relatively weak compared to environmental exposures and lifestyle factors. In our data, we did find that BMI at age of 20 years was inversely correlated with age at menarche, suggesting that age at menarche be influenced both by genetic and lifestyle factors. The genetic risk score for menopause (GRS2) was not associated with disease risk. Recently, genetic risk scores have been used to measure combined genetic effects on cancer risk^27^,^29^,^30^. Early age at menarche and late age at natural menopause are established risk factors for endometrial cancer. It is plausible that SNPs related to age at menarche or age at natural menopause may also influence cancer risk through their influence on menarche and menopause. Our results demonstrated that GRS1, but not GRS2, was associated with endometrial cancer risk. These results were similar to those of a meta-analysis in which data from six population-based studies were pooled and analyzed. Compared to individuals in the lowest quintile, women in the highest quintiles (4^th^ and 5^th^) of the genetic risk score on age at menarche had odds ratios of 1.14 (95% CI, 1.01 to 1.28) and 1.13 (95% CI, 1.00 to 1.27), respectively, for breast cancer^27^. These observations suggest that estrogen exposure at early ages may be more hazardous to the target tissue than exposures at later ages, which is consistent with the notion that breast tissue is more sensitive to carcinogens such as ionizing radiation when the tissue is young or at an early stage of development. Of course, longer exposure may also be a possible explanation for the association with early age menarche.

In addition to TNMC, we also calculated total months of menstrual cycles (TMMC) during life or to the time of study, and analyzed its association with endometrial cancer risk. Like TNMC, TMMC was also significantly associated with endometrial cancer risk (data not shown). Given that TMMC was quite similar to TNMC and that average length of menstrual cycle was not associated with the disease, we did not present the results of TMMC analysis in this report. Although an estrogen effect is the major underlying mechanism explaining the findings of our analysis, estrogen levels and the balance between estrogen and progesterone may not be constant in each menstrual cycle, especially when the cycles are anovulatory. Secondary amenorrhea, which was not considered in our TNMC calculation, may also be a potential factor influencing our assessment of estrogen effect. Furthermore, the reproductive and menstrual parameters used for calculation of the composite variables may have other biological effects which are not estrogenic or have additional effects which are independent from their contributions to the composite variables. Thus, caution should be exercised when one interprets our results.

Obesity is a strong risk factor for endometrial cancer. Obese women have been reported to have 4 to 6-fold increases in endometrial cancer risk compared to women with normal weight^31^,^32^. Obesity may explain 40% of endometrial cancer risk in affluent societies^5^,^33^. Long-term overweight and substantial weight gain in early adulthood are also related to the risk of endometrial cancer^34^,^35^,^36^,^37^. In our study, we have observed similar associations between obesity and risk of endometrial cancer^38^. Obesity’s link to endometrial cancer has been attributable in part to its influence on estrogen^39^,^40^. Obesity increases estrogen levels in postmenopausal women and reduces progesterone levels in premenopausal women^41^. To rule out or minimize the influence of obesity on the associations that we found, we adjusted for BMI in the logistic regression models. Our results remained significant for the composite measure of reproductive features, but not for GRS1. These findings suggest that the estrogenic effect from menstrual and reproductive factors may be independent of obesity. The risk effect of genetic determinants is weak and less independent of obesity. We also evaluated risk associations with the composite reproductive measure and genetic score more specifically by restricting our analysis to subgroups of women stratified by BMI categories. Interestingly, these analyses indicated that the associations were mainly seen among overweight women. Normal weight and obese women did not show any associations between these composite variables and endometrial cancer risk. The observations raise questions regarding the possible interaction between obesity and other estrogen-related risk factors in endometrial cancer, but before any conclusion can be drawn we need to confirm these findings in additional studies with large sample size. In the study, we also found a possible synergy between the composite reproductive measure and genetic risk score for age at menarche in association with endometrial cancer risk. This synergy was attenuated slightly when BMI was included in the analysis, but remained statistically significant. However, statistically significant interaction between TNMC and GRS for age at menarche was not observed in the logistic regression model.

Finally, the results of our study should be interpreted with caution because there are a number of inherited methodological limitations. First, the study results were established on the basis of single-site investigation with a moderate sample size and inherited possibility for selection bias. Second, some of the exposure variables in the study were subject to possible recall bias, though the likelihood and impact appeared to be small. Third, these observations need to be confirmed by independent studies. Fourth, in calculation of TNMC, we did not consider the potential impact of secondary amenorrhea that some women might experience occasionally.

In conclusion, our study suggests that high frequency of menstrual cycles, resulting from long reproductive life, fewer pregnancies and other factors, may play a role in the etiology of endometrial cancer. Furthermore, this composite measure appears to have synergistic interactions with common genetic polymorphisms that determine early age at menarche, and the synergy is independent of obesity, a strong risk factor for endometrial cancer. If our findings can be confirmed by other epidemiologic studies, we may consider including the composite measure in risk prediction models for endometrial cancer.

## Methods

### Study Population

A population-based case-control study was conducted in Connecticut between December 2004 and March 2009. The study was approved by the Institutional Review Boards at Yale University (IRB number: HIC 0305025270), Connecticut State Department of Health and 28 Connecticut hospitals involved. The methods were carried out in accordance with the approved guidelines. Eligibility of the study subjects has been described elsewhere^38^. Patients with primary endometrial cancer newly diagnosed between October 2004 and September 2008 were identified through the Rapid Case Ascertainment Shared Resource of the Yale Cancer Center (RCA). Control women, frequency matched to cases on age group (35–51, 52–59, 60–64, 65–69, 70–74, and 75–79 years), were recruited through pre-letter assisted random-digit residential telephone dialing. After signing a written informed consent, each study participant provided a blood sample and underwent an in-person interview using a structured questionnaire which ascertained information on demographic features, menstrual and reproductive history, use of exogenous hormones, oral contraceptives (OC) use, medical history, family history of cancer, physical activity, anthropometric measurements, as well as tobacco smoking, alcohol drinking and dietary habits. Subjects with a history of prior cancer diagnosis were excluded from the study.

### Total Number of Menstrual Cycles

To evaluate the collective effect of reproductive factors, we constructed a total number of menstrual cycles variable (TNMC), which estimated the total number of menstrual cycles experienced during life or up to the time of our investigation. This composite measure was computed from age at menarche, age at menopause, average length of menstrual cycles, total months of all pregnancies, and total months of breastfeeding. TNMC was calculated based on the following formula: [(age at menopause − age at menarche) × 12 − total months of all pregnancies − number of live births × 1.5 − (total months of breastfeeding)/2 − total months of oral contraceptive use] × 30/average length of menstrual cycles (days). Menstrual cycles were assumed to resume at 1.5 months post parturition if no breastfeeding was performed^15^. If breastfeeding occurred after parturition, we assumed that menstrual cycles resumed on average at 1.5 months plus half of the duration of breastfeeding for each such parturition. Partial or full breastfeeding was not considered in the calculation. For premenopausal women, age at endometrial cancer diagnosis (cases) or study interview (controls) was used as a surrogate for “age at menopause” in calculation of TNMC.

### Genotype and Genetic Risk Scores

Genomic DNA was isolated from peripheral blood using a commercial DNA extraction kit. SNP genotyping was performed using the HumanOmniExpress BeadChips (Illumina Inc, San Diego, CA). The BeadChips results were run on an Illumina iScan system using the Infinium HD Assay Super Automated Protocol. The GenomeStudio Genotyping (GT) Module (Illumina Inc, San Diego, CA) was used for data normalization and genotype calling. After filtering out SNPs with completion rates <90%, minor allele frequencies <1%, and not in Hardy–Weinberg equilibrium (P < 0.0001) among the controls, 1053 subjects including 482 cases and 571 controls with genotype information on 649,351 SNPs were included in a previous genome-wide association study (GWAS) of endometrial cancer^17^. This information was used for our construction of genetic risk score (GRS). Two types of GRS were calculated. One was non-weighted GRS which was the total number of risk alleles among all the SNPs selected for GRS calculation. For each selected SNP, we assigned 0, 1, or 2 to indicate the number of risk alleles, assuming an equal and additive effect of each risk allele. The other index was a weighted GRS that was the sum of the product of risk allele number and beta coefficient obtained from published GWAS results of each SNP. Alleles associated with younger age at menarche or older age at natural menopause were defined as risk alleles.

Several GWAS have been published reporting SNPs associated with age at menarche and age at natural menopause. We selected 26 menarche-associated^18^,^19^,^20^,^21^,^22^ and 22 menopause-associated SNPs^23^,^24^,^25^,^26^ to construct two Genetic Risk Scores (GRS) for our study, one for menarche (GRS1) and one for menopause (GRS2). The criteria for SNP selection included: (a) linkage disequilibrium between SNPs in the same gene was low (R^2^ < 0.5); (b) number of failed genotypes for each SNP was fewer than five study subjects; and (c) associations between SNPs and menarche or menopause age had the same direction between our study and the GWAS. The few study subjects that had missing SNP genotype information were assigned mean risks that were calculated separately in 482 cases and 571 controls, rounded to the nearest whole unit. For the 26 SNPs that were not genotyped in our data, highly linked proxy SNPs were selected using the 1000 Genome Pilot1 for the CEU population, which is primarily of Northern/Western European ancestry ( https://www.broadinstitute.org/mpg/snap/ldsearch.php).

### Obesity Variable

Body mass index (BMI, kg/m^2^) was calculated based on self-reported weight and height 5 years before interview. BMI was classified into three groups: normal weight (<25 kg/m^2^), overweight (25 < 30 kg/m^2^), and obese (≥30 kg/m^2^). In addition to the cross-sectional evaluation, we also calculated BMI change over time. BMI change was defined as [(BMI 5 years before interview − BMI at 20 s)/BMI at 20 s] × 100, and the values were grouped into four categories: BMI change less than or equal to 5%, BMI change greater than 5% but less than or equal to 20%, BMI change greater than 20% but less than or equal to 35%, and BMI change greater than 35%.

### Statistical Analysis

Both continuous and categorical variables were used for data analysis of total numbers of menstrual cycles and genetic risk scores. For categorical data, TNMC was classified into four groups according to the quartile distribution among control subjects. GRS was divided into three groups based on tertile distributions in the controls. Attained age, age at menarche, and age at natural menopause were considered as continuous variables. Education was grouped into 4 levels, including less than 12 years, 12 years to 3 years of college, completion of college or university, and graduate school. Menopausal status was categorized as pre-menopause, natural post-menopause and induced post-menopause. Linear regression was used to analyze the associations of GRS with age at menarche and age at natural menopause, with adjustment for age and race. Differences of continuous variables between cases and controls were evaluated by Student t statistic. Associations of endometrial cancer risk with TNMC and GRS were analyzed using the unconditional logistic regression that yielded odds ratios and 95% confidence intervals. Both unadjusted and adjusted models were developed in the analysis, and various multivariable models were created to adjust as appropriate for sets of variables that included age, race, years of education, BMI, BMI change, estrogen hormone use, family history of cancer and OC use. Trends were examined by considering odds ratios for continuous variables. Associations between SNPs and endometrial cancer risk were evaluated by log-additive logistic regression using PLINK (version 1.07; http://pngu.mgh.harvard.edu/~purcell/plink/). All other statistical tests were performed with Statistical Analysis System (SAS) software (version 9.2, SAS Institute Inc., Cary, NC). All P-values were two-sided.

## Additional Information

**How to cite this article**: Wang, Z. *et al.* Joint Effect of Genotypic and Phenotypic Features of Reproductive Factors on Endometrial Cancer Risk. *Sci. Rep.* **5**, 15582; doi: 10.1038/srep15582 (2015).

## Supplementary Material

Supplementary Information

## Figures and Tables

**Table 1:** Distributions of study variables in endometrial cancer patients and controls.

Categorical Variable	Case (n = 482) No. (%)	Control (n = 571) No. (%)	*P* value
Race			0.554
Non white	5(1.04)	4(0.70)	
White	477(98.96)	567(99.30)	
BMI (kg/m^2^)^			
<25	96(21.57)	226(47.48)	10^−26.8^
25-<30	110(24.72)	154(32.35)	
≥30	239(53.71)	96(20.17)	
BMI change			10^−8.4^
≤5%	9(2.32)	18(4.64)	
>5%-≤20%	76(19.59)	134(34.45)	
>20%-≤35%	102(26.29)	119(30.59)	
>35%	201(51.80)	118(30.33)	
Education			10^−4.2^
<12 yrs.	173(35.89)	150(26.27)	
12 yrs.-3 yrs. College	125(25.93)	122(21.37)	
College/university	92(19.09)	143(25.04)	
Graduate school	92(19.09)	156(27.32)	
Menopause status			10^−7.4^
Premenopause	60(12.71)	104(18.67)	
Natural menopause	344(72.88)	428(76.84)	
Induced menopause	68(14.41)	25(4.49)	
Estrogen use			0.074
Yes	150(31.91)	201(37.29)	
No	320(68.09)	338(62.71)	
OC use			0.010
Ever	277(57.83)	368(65.60)	
Never	202(42.17)	193(34.40)	
Family history of cancer			0.24
Yes	324(67.22)	364(63.75)	
No	158(32.78)	207(36.25)	
Numerical Variable	Mean ± SD	Mean ± SD	
Age (year)	60.55 ± 9.18	61.84 ± 11.12	0.053
Age at menarche (year)	12.30 ± 1.54	12.64 ± 1.60	0.005
Age at natural menopause (year)*	50.91 ± 5.09	50.77 ± 4.15	0.68
Total numbers of menstrual cycles	422.9 ± 101.0	396.1 ± 106.4	10^−4.3^
Total pregnant months	23.13 ± 12.27	24.78 ± 13.28	0.060
GRS1	27.90 ± 3.78	27.36 ± 3.82	0.021
Weighted GRS1	2.09 ± 0.04	2.03 ± 0.37	0.011
GRS2*	21.43 ± 2.75	21.42 ± 2.68	0.97
Weighted GRS2*	6.343 ± 1.04	6.337 ± 1.01	0.92

^*^Subjects with induced menopause were excluded (344 cases and 428 controls).

^^^BMI—5 years before interview.

**Table 2:** Linear regression between GRS and age at menarche or age at natural menopause among controls.

Genetic risk score (GRS)	β^a^	95% CI		*P*^b^
Age at menarche				
GRS1^c^				
<27	0	Reference		
27-<30	−0.14	−0.46	0.19	0.33
≥30	−0.39	−0.71	−0.07	0.044
Continuous	−0.27	−0.47	−0.08	0.007
Weighed GRS1				
<1.86	0	Reference		
1.86-<2.19	−0.08	−0.31	0.17	0.55
≥2.19	−0.27	−0.51	−0.04	0.024
Continuous	−0.40	−0.66	−0.14	0.002
Age at natural menopause				
GRS2^d^				
<21	0	Reference		
21-<24	0.87	0.16	1.59	0.016
≥24	2.16	1.31	3.01	10^−6^
Continuous	0.25	0.13	0.37	10^−4.4^
Weighed GRS2^d^				
<5.85	0	Reference		
5.85-<6.74	0.66	−0.12	1.44	0.0002
≥6.74	1.49	0.71	2.27	0.095
Continuous	0.57	0.26	0.89	0.0004

^a^Mean difference in age at menarche or age at natural menopause.

^b^Adjusted for age and race.

^c^GRS1 was calculated based on the 26 SNPs in Table 2.

^d^GRS2 was calculated based on the 22 SNPs in Table 2.

**Table 3:** Associations of endometrial cancer with total numbers of menstrual cycles and genetic risk scores on menarche and menopause among all subjects.

Variable	Case/Control	OR^a^	95%CI		OR^b^	95%CI		OR^c^	95%CI		OR^d^	95%CI		OR^e^	95%CI	
TNMC																
Continuous*	459/538	**1.29**	**1.14**	**1.46**	**1.40**	**1.22**	**1.60**	**1.33**	**1.15**	**1.53**	**1.28**	**1.10**	**1.50**	**1.27**	**1.07**	**1.50**
≤411.5	173/269	1.00			1.00			1.00			1.00			1.00		
>411.5	286/269	**1.65**	**1.28**	**2.13**	**1.86**	**1.42**	**2.43**	**1.72**	**1.29**	**2.28**	**1.52**	**1.10**	**2.09**	**1.49**	**1.07**	**2.08**
GRS1																
Continuous	482/571	**1.04**	**1.01**	**1.07**	**1.04**	**1.00**	**1.07**	**1.03**	**1.00**	**1.07**	1.01	0.97	1.05	1.01	0.97	1.05
≤27	216/266	1.00			1.00			1.00			1.00			1.00		
>27	296/275	**1.33**	**1.04**	**1.69**	**1.30**	**1.01**	**1.66**	**1.29**	**1.00**	**1.68**	1.13	0.85	1.50	1.06	0.79	1.43
GRS2																
Continuous	414/546	1.00	0.96	1.05	1.00	0.95	1.05	1.01	0.96	1.07	1.01	0.96	1.07	1.00	0.94	1.06
≤21	212/275	1.00			1.00			1.00			1.00			1.00		
>21	202/271	0.97	0.75	1.25	0.98	0.75	1.27	1.01	0.77	1.33	1.06	0.79	1.43	0.97	0.71	1.33

^*^100 menstrual cycles per unit of increment.

^a^no adjustment.

^b^adjusted for age, race, and education.

^c^adjusted for age, race, education, family cancer of history, OC use, and estrogen.

^d^adjusted for age, race, education, family cancer of history, OC use, estrogen use, and BMI.

^e^adjusted for age, race, education, family cancer of history, OC use, estrogen use, and BMI change.

**Table 4:** Associations of endometrial cancer with total numbers of menstrual cycles and genetic risk scores on menarche and menopause among women who had natural menopause.

Variable	Case/Control	OR^a^	95%CI		OR^b^	95%CI		OR^c^	95%CI		OR^d^	95%CI		OR^e^	95%CI	
TNMC																
Continuous*	330/415	**1.28**	**1.09**	**1.51**	**1.32**	**1.11**	**1.57**	**1.24**	**1.04**	**1.49**	1.16	0.95	1.43	**1.21**	**1.00**	**1.46**
≤424	132/208	1.00			1.00			1.00			1.00			1.00		
>424	198/207	**1.51**	**1.13**	**2.02**	**1.60**	**1.18**	**2.17**	**1.44**	**1.05**	**1.99**	1.25	0.87	1.79	**1.40**	**1.01**	**1.95**
GRS1																
Continuous	344/428	1.02	0.99	1.06	1.01	0.98	1.05	1.02	0.98	1.06	1.00	0.96	1.04	1.01	0.97	1.05
≤27	158/216	1.00			1.00			1.00			1.00			1.00		
>27	186/212	1.20	0.90	1.59	1.14	0.85	1.52	1.19	0.88	1.60	1.11	0.79	1.54	1.12	0.82	1.52
GRS2																
Continuous	344/428	1.01	0.95	1.06	1.00	0.95	1.06	1.00	0.95	1.06	1.00	0.94	1.06	0.99	0.94	1.05
≤22	226/280	1.00			1.00			1.00			1.00			1.00		
>22	118/148	0.99	0.73	1.33	0.93	0.68	1.26	0.92	0.67	1.25	0.94	0.66	1.33	0.92	0.67	1.27

^*^100 menstrual cycles per unit of increment.

^a^no adjustment.

^b^adjusted for age, race, and education.

^c^adjusted for age, race, education, family cancer of history, OC use, and estrogen use.

^d^adjusted for age, race, education, family cancer of history, OC use, estrogen use, and BMI.

^e^adjusted for age, race, education, family cancer of history, OC use, estrogen use, and BMI change.

**Table 5:** Joint effects of total numbers of menstrual cycles and genetic risk score of menarche on endometrial cancer risk.

TNMC/GRS1	Case/Control	OR^a^	95%CI		OR^b^	95%CI		OR^c^	95%CI		OR^d^	95%CI		OR^e^	95%CI	
Low/Low	69/138	1.00			1.00			1.00			1.00			1.00		
Low/High	104/131	**1.59**	**1.08**	**2.34**	**1.57**	**1.06**	**2.33**	**1.45**	**0.97**	**2.19**	1.28	0.81	2.03	0.98	0.59	1.61
High/Low	132/139	**1.90**	**1.31**	**2.76**	**2.13**	**1.44**	**3.15**	**1.90**	**1.26**	**2.85**	**1.69**	**1.07**	**2.66**	**1.38**	**0.85**	**2.23**
High/High	130/154	**2.37**	**1.63**	**3.44**	**2.66**	**1.81**	**3.93**	**2.34**	**1.56**	**3.51**	**1.80**	**1.15**	**2.84**	**1.57**	**0.97**	**2.53**
P for interaction			0.35			0.40			0.55			0.56			0.64	

^a^no adjustment.

^b^adjusted for age, race, and education.

^c^adjusted for age, race, education, family cancer of history, OC use, and estrogen use.

^d^adjusted for age, race, education, family cancer of history, OC use, estrogen use, and BMI.

^e^adjusted for age, race, education, family cancer of history, OC use, estrogen use, and BMI change.
