# Supplementary material for: Joint Effect of Genotypic and Phenotypic Features of Reproductive Factors on Endometrial Cancer Risk
Source: Sci Rep. 2015 Oct 26;5:15582. doi: 10.1038/srep15582 (PMC4620445; doi:10.1038/srep15582)
Supplement: Supplementary Information [file srep15582-s1.pdf]

Joint Effect of Genotypic and Phenotypic Features of Reproductive Factors on Endometrial Cancer Risk

Zhanwei Wang[1], Harvey Risch[2], Lingeng Lu[2], Melinda L. Irwin[2], Susan Mayne[2, *], Peter Schwartz[3], Thomas Rutherford[3], Immaculata De Vivo[4, 5], Herbert Yu[1, **]

[1] Cancer Epidemiology Program, University of Hawaii Cancer Center, Honolulu, HI

[2] Department of Chronic Disease Epidemiology, Yale School of Public Health, New Haven, CT, and Yale Cancer Center

[3] Department of Obstetrics and Gynecology, Yale School of Medicine, New Haven, CT, and Yale Cancer Center

[4] Channing Division of Network Medicine, Department of Medicine, Brigham and Women's Hospital and Harvard Medical School, Boston, MA 02115, USA

[5] Department of Epidemiology, Program in Genetic Epidemiology and Statistical Genetics, Harvard School of Public Health, Boston, MA 02115, USA

* Current affiliation: Center for Food Safety and Applied Nutrition, Food and Drug Administration, College Park, MD 20740, USA

Supplementary Table S1. Menarche- and menopause-associated SNPs used for construction of genetic risk scores (GRS)

Age at menarche					Age at natural menopause				
CHR[1]	GWAS SNP	Proxy SNP[2]	D'[3]	Within or nearest gene	CHR[1]	GWAS SNP	Proxy SNP[2]	D'[3]	Within or Nearest gene
1	rs633715			SEC16B	1	rs4246511	rs4414011	1.000	RHBDL2
2	rs17268785	rs17047854	1.000	CCDC85A	1	rs7528241			CDC73
2	rs17188434	rs4369815	1.000	NR4A2	1	rs1635501	rs735943	1.000	EXO1
2	rs12617311	rs2048968	1.000	PLCL1	2	rs2303369	rs704795	1.000	FNDC4
3	rs6762477	rs1061474	1.000	RBM6	2	rs10183486	rs930036	0.963	TLK1
3	rs7642134			VGLL3	5	rs890835	rs1978604	1.000	RNF44
3	rs6439371			TMEM108, NPHP3	6	rs2153157			SYCP2L
3	rs2002675	rs10049246	0.812	TRA2B, ETV5	6	rs1046089			BAT2
5	rs13187289	rs329119	1.000	PHF15	8	rs2517388			ASH2L
5	rs2348186	rs2348188	1.000	SPOKEN	10	rs11597068			RPS26P39,BUB3
5	rs7701979			SPOKEN	11	rs12294104	rs10835646	1.000	MPPED2;C11orf46
6	rs7759938			LIN28B	12	rs2277339			PRIM1
6	rs314277			LIN28B	13	rs3736830	rs2146143	1.000	KPNA3
6	rs364663	rs314272	1.000	LIN28B	13	rs7333181			ARHGEF7
6	rs1361108	rs4422634	1.000	C6orf173, TRMT11	15	rs6495785			ATPBD4-AS1
7	rs1079866	rs4720408	0.692	INHBA	15	rs2307449	rs2302084	1.000	POLG
9	rs2090409	rs7862517	0.954	TMEM38B	16	rs10852344	rs11075032	0.966	TNFRSF17; GSPT1
9	rs4452860	rs13300395	1.000	NR[4]	19	rs12611091			BSK1
9	rs7861280	rs2417687	1.000	NR[4]	19	rs1551562			BSK1
11	rs4929923			TRIM66	19	rs11668344	rs11668309	1.000	TMEM150B
11	rs10899489	rs10793311	1.000	GAB2/ZNF75C	19	rs12461110			NLRP11
11	rs6589964			BSX	20	rs236114			MCM8
14	rs6575793	rs4076891	1.000	BEGAIN					
16	rs9939609	rs3751812	1.000	FTO					
16	rs1364063			NFAT5					
17	rs9635759			CA10					

1. CHR: chromosome.
2. Proxy SNP: SNPs used for GRS calculation from our genotyping dat.
3. D': Linkage disequilibrium.
4. Not reported.

Supplementary Table S2. Associations of endometrial cancer with total numbers of menstrual cycles or genetic risk scores on menarche or menopause among 322 women with BMI<25

Variable	OR[a]	95%CI		OR[b]	95%CI		OR[c]	95%CI	
**TNMC**									
Continuous*	1.06	0.84	1.34	1.19	0.92	1.54	1.14	0.87	1.51
≤411.5	1.00			1.00			1.00		
>411.5	0.99	0.61	1.62	1.17	0.70	1.95	1.09	0.62	1.89
**GRS1**									
Continuous	1.01	0.94	1.07	1.01	0.94	1.08	1.00	0.94	1.07
≤27	1.00			1.00			1.00		
>27	0.88	0.55	1.42	0.93	0.57	1.52	0.86	0.52	1.43
**GRS2**									
Continuous	1.03	0.93	1.13	1.04	0.94	1.15	1.03	0.93	1.13
≤21	1.00			1.00			1.00		
>21	0.99	0.58	1.72	1.03	0.59	1.80	1.16	0.67	2.01

* 100 menstrual cycles per unit of increment

a. no adjustment.

b. adjusted for age, race, and education.

c. adjusted for age, race, education, family cancer of history, OC use, and estrogen use.

Supplementary Table S3. Associations of endometrial cancer with total numbers of menstrual cycles or genetic risk scores on menarche or menopause among 264 women with BMI between 25 and 30

Variable	OR[a]	95%CI		OR[b]	95%CI		OR[c]	95%CI	
**TNMC**									
Continuous*	**1.51**	**1.14**	**1.98**	**1.61**	**1.19**	**2.16**	**1.55**	**1.13**	**2.12**
≤411.5	1.00			1.00			1.00		
>411.5	**2.57**	**1.51**	**4.39**	**2.63**	**1.50**	**4.64**	**2.48**	**1.37**	**4.50**
**GRS1**									
Continuous	1.04	0.97	1.10	1.02	0.95	1.09	1.02	0.96	1.09
≤27	1.00			1.00			1.00		
>27	**1.81**	**1.10**	**2.96**	**1.70**	**1.02**	**2.83**	1.66	0.98	2.83
**GRS2**									
Continuous	1.05	0.95	1.15	1.04	0.94	1.14	1.04	0.94	1.16
≤21	1.00			1.00			1.00		
>21	1.46	0.86	2.50	1.39	0.80	2.42	0.97	0.56	1.70

* 100 menstrual cycles per unit of increment

a. no adjustment.

b. adjusted for age, race, and education.

c. adjusted for age, race, education, family cancer of history, OC use, and estrogen use.

Supplementary Table S4. Associations of endometrial cancer with total numbers of menstrual cycles or genetic risk scores on menarche or menopause among 335 women with BMI>30

Variable	OR[a]	95%CI		OR[b]	95%CI		OR[c]	95%CI	
**TNMC**									
Continuous*	1.11	0.89	1.40	1.26	0.98	1.62	1.20	0.92	1.57
≤411.5	1.00			1.00			1.00		
>411.5	1.11	0.68	1.83	1.37	0.81	2.33	1.25	0.71	2.18
**GRS1**									
Continuous	0.99	0.93	1.05	0.99	0.93	1.06	1.00	0.94	1.06
≤27	1.00			1.00			1.00		
>27	1.05	0.65	1.70	1.09	0.66	1.78	1.12	0.68	1.85
**GRS2**									
Continuous	0.96	0.88	1.05	0.96	0.88	1.06	0.97	0.88	1.07
≤21	1.00			1.00			1.00		
>21	0.92	0.54	1.54	0.94	0.55	1.59	0.99	0.60	1.66

* 100 menstrual cycles per unit of incremen[illegible]

a. no adjustment.

b. adjusted for age, race, and education.

c. adjusted for age, race, education, family cancer of history, OC use, and estrogen use.
